# Immune markers for pulmonary aspergillosis in patients with chronic obstructive pulmonary disease: a narrative review

**DOI:** 10.3389/fimmu.2025.1687790

**Published:** 2025-10-23

**Authors:** Yuke Hu, Xingbo Wang, Hao Wu, Haibo Deng, Fei Gao, Yonghong Wu

**Affiliations:** ^1^ School of Medical and Life Sciences, Chengdu University of Traditional Chinese Medicine, Chengdu, China; ^2^ Department of Respiratory Medicine, Jianyang People’s Hospital, Jianyang, China; ^3^ School of Modern Chinese Medicine Industry/School of Pharmacy, Chengdu University of Traditional Chinese Medicine, Chengdu, China

**Keywords:** chronic obstructive pulmonary disease, pulmonary aspergillosis, immune biomarkers, galactomannan, diagnostic algorithm

## Abstract

COPD complicated by pulmonary aspergillosis (COPD-PA) encompasses invasive, chronic, and allergic phenotypes and is increasingly recognized as a high-burden comorbidity. Clinical recognition is often hindered by nonspecific manifestations, corticosteroid-suppressed inflammatory signs, and the suboptimal performance of serum galactomannan in non-neutropenic hosts. To define the translational utility of immune biomarkers in this population, evidence was synthesized from international guidelines and contemporary studies in COPD-enriched cohorts, and performance was appraised across diagnostic, monitoring, and prognostic domains. Convergent findings indicate that bronchoalveolar-lavage galactomannan facilitates early diagnosis; serum galactomannan indices stratify risk during exacerbations; Aspergillus-specific IgG supports rule-in for invasive and chronic disease; and pentraxin-3 adds prognostic information. Cytokines central to COPD-PA pathobiology, including interleukin-1β, interleukin-6, interleukin-8, and interleukin-17, provide adjunctive diagnostic value, whereas (1→3)-β-D-glucan shows limited specificity. On this basis, a three-tier framework was developed that classifies biomarkers as clinically validated, mechanistically promising, or exploratory, and this framework was translated into subtype-tailored panels and decision rules that favor either-positive criteria for screening and both-positive criteria for confirmation. It is concluded that immune biomarkers complement microbiology and imaging, expand access when bronchoscopy or biopsy is not feasible, and enable longitudinal risk stratification. Priorities include COPD-specific thresholds, assay standardization, and multicenter validation, with particular emphasis on chronic pulmonary aspergillosis and allergic bronchopulmonary aspergillosis. Biomarker-guided immunomodulation may benefit selected phenotypes but requires rigorous evaluation before clinical adoption.

## 1 Introduction

Chronic obstructive pulmonary disease with pulmonary aspergillosis (COPD–PA) refers to COPD complicated by infections across the *Aspergillus* spectrum—primarily invasive pulmonary aspergillosis (IPA) (1), chronic pulmonary aspergillosis (CPA) (2), and allergic bronchopulmonary aspergillosis (ABPA) (3). The clinical burden of COPD–PA is considerable. A 2020 global systematic review estimated that 1.3–3.9% of hospitalized COPD patients develop invasive aspergillosis annually, based on studies conducted across multiple regions from 2000 to 2019 (4). Numerous studies have highlighted that COPD–PA significantly exacerbates outcomes by accelerating the decline in lung function, diminishing quality of life, and increasing mortality (5–7). Diagnosing COPD–PA in clinical practice presents several challenges: COPD symptoms often obscure aspergillosis, and the clinical manifestations of aspergillosis are nonspecific (8–11); corticosteroid exposure can suppress fever and inflammatory responses (12); the sensitivity of galactomannan (GM) is lower in non-neutropenic COPD patients than in classic immunocompromised individuals (13).

In recent years, immune markers have attracted growing interest in COPD–PA, including GM, *Aspergillus*-specific IgG (Asp IgG), C-reactive protein (CRP), and pentraxin-3 (PTX3) (14–17). The immunopathogenesis of COPD–PA is complex; nevertheless, the pursuit of immune markers remains feasible. COPD-related epithelial injury (18), impaired mucociliary clearance (19), neutrophil/macrophage dysfunction (20, 21), and skewed T-cell responses create a permissive environment for Aspergillus persistence and overgrowth, thereby providing a biological rationale for early diagnosis, prognostic assessment, and therapeutic stratification (22).

Although interest in COPD–PA has been growing, dedicated investigations remain limited, with most studies treating COPD and PA as separate entities. To the best of our knowledge, no review has yet consolidated immune markers specific to COPD–PA across IPA, CPA, and ABPA. In this review, we analyze immune dysregulation in COPD–PA, synthesize the current evidence, and propose an evidence-tiered marker framework specifically tailored to COPD–PA, aimed at guiding research priorities and facilitating its translational applications.

## 2 Immune dysregulation in COPD–PA

Chronic inflammation in COPD damages airway and lung structures, thereby impairing the respiratory tract’s natural defenses and promoting *Aspergillus* colonization and invasion (23). Moreover, treatments commonly used for COPD, such as inhaled corticosteroids (ICS) and broad-spectrum antibiotics, can exacerbate immune dysregulation and impair the clearance of pulmonary *Aspergillus* infections (24). Following infection, fungal virulence factors directly damage airway epithelial cells (25). Concurrently, excessive secretion of pro-inflammatory cytokines and chemokines by immune cells further exacerbates immune dysregulation, ultimately worsening COPD. Importantly, this entire pathogenic cycle is inextricably associated with immune dysregulation.

### 2.1 Adhesion as the initial step in infection: epithelial cells as the first line of defense against *Aspergillus*

*Aspergillus* species are ubiquitous in the environment, and most *Aspergillus* conidia are likely cleared from the airway by the ciliary action of the airway epithelium, making the inhalation of conidia rarely cause disease in immunocompetent hosts (19, 26). In addition to ciliary action, specialized epithelial secretory cells prevent spore colonization by secreting mucus (27). Human bronchial epithelial cells, when exposed to *Aspergillus fumigatus (A. fumigatus)* conidia *in vitro*, inhibit conidial germination through FleA recognition, thereby reducing fungal invasiveness (28). However, in COPD patients, factors such as cigarette smoke and recurrent infections compromise epithelial function, impair conidial clearance, and increase susceptibility to *Aspergillus* infection (18). For instance, inhalation of cigarette smoke, the primary risk factor for COPD (29), induces epithelial injury and triggers the release of damage-associated molecular patterns (DAMPs) (30).

Adherence to the epithelium is the first step in infection development, mediated by multiple receptor-ligand interactions between *Aspergillus* and the epithelium, including pattern-recognition receptors (PRRs) recognizing GM and (1→3)-β-D-glucan (BDG), among others. Epithelial PRRs—including Toll-like receptors (TLRs), C-type lectin receptors (CLRs), and NOD-like receptors (NLRs)—can activate innate inflammatory cascades. These receptors detect DAMPs that initiate sterile inflammation, as well as persistent fungal pathogen-associated molecular patterns (PAMPs), especially GM and BDG, that sustain chronic inflammation (31, 32). The relationship between COPD and PA is bidirectional, persistent *Aspergillus* drives excessive immune responses that exacerbate airway inflammation and tissue damage in COPD (33). *A. fumigatus*-derived extracellular polysaccharides and gliotoxins directly disrupt epithelial integrity (34), impairing airway repair in COPD.

TLRs, including TLR2 and TLR4, are critical components of the innate immunity in COPD–PA. A study has shown that through airway brushings, the mRNA expression of TLR2 and TLR4 in the airway epithelial cells of patients with mild-to-moderate COPD was increased by approximately 2.4-fold and 8.7-fold, and their expression is also increased in severe COPD (35). This increase is likely associated with the chronic inflammatory processes underlying COPD. In lung parenchyma biopsy samples, mRNA expression of TLR2 and TLR4 was elevated in mild-to-moderate COPD, but reduced in severe cases, possibly related to bacterial infection and to protective responses that limit oxidative stress in lung tissue (35, 36). Ou et al. demonstrated that cigarette smoke activates the TLR4–MyD88–NF-κB signaling cascade (37), and cigarette smoke is the primary risk factor for COPD (29). In COPD, TLR expression is upregulated in most settings, thereby amplifying airway inflammation. TLR2 and TLR4 mediate multiple immune responses during *Aspergillus* infection (38, 39), with TLR2 inducing Dectin-1 expression and recognizing BDG to activate the SYK/CARD9–NF-κB and RAF-1 pathways, releasing pro-inflammatory cytokines interleukin-6 (IL-6) and interleukin-8 (IL-8), as well as C-X-C motif chemokine ligand 1 (CXCL1) and C-X-C motif chemokine ligand 2 (CXCL2), and promoting neutrophil recruitment (40). *In vitro* stimulation of lung epithelial cells with *A. fumigatus* conidia demonstrated that loss of function of TLR2 or Dectin-1 results in reduced production of inflammatory cytokines (41).

TLR/MyD88 activation not only activates NF-κB but also activates the JNK pathway in the MAPK signaling cascade, leading to the activation of the AP-1 transcription factor (42, 43). These downstream transcription factors regulate inflammation, immune cell recruitment, and pathogen clearance (44, 45). JNK pathway activation correlates with epithelial damage and airway inflammation in COPD (46, 47). Cui et al. found that stimulation of human bronchial epithelial BEAS-2B cells with *A. fumigatus* conidia activates the MAPK-JNK pathway, leading to the upregulation of IL-27 and tumor necrosis factor-alpha (TNF-α); The JNK inhibitor SP600125 significantly attenuated this cytokine production (48). These findings indicate that TLRs, Dectin-1, and JNK are likely involved in the pathophysiological processes of COPD–PA. Their downstream products promote infection defense but may also aggravate airway inflammation in COPD.

### 2.2 The phagocytic system as the second line of host defense

Failure of mucociliary clearance enables *Aspergillus* conidia to adhere to and potentially colonize the epithelial surface (49). This event activates downstream immune effectors through multiple signaling pathways (outlined earlier), thereby initiating the host’s second line of defense. The second line of defense includes alveolar macrophages (AMs), which are crucial in destroying the conidia of *Aspergillus*, as well as neutrophils that kill fungal hyphae and germinating spores (50).

Macrophages perform multiple interrelated functions crucial to pulmonary host defense, including phagocytosis of inhaled particles and pathogens (e.g., cigarette smoke particulates and *Aspergillus* conidia), immune surveillance via PRRs, antigen processing and presentation to prime adaptive responses, and orchestration of inflammatory signaling (51). Excessive oxidative stress (e.g., glutathione-mediated) impairs phagocytosis, permitting cytotoxic release from necrotic cells, a dysregulation that exacerbates COPD inflammation (52). Consistently, elevated oxidative stress in COPD is associated with diminished bacterial phagocytosis, linked to mitochondrial dysfunction and the accumulation of intracellular oxidants (53). COPD macrophages significantly increase the expression of TNF-α, IL-1β, and IL-6 upon exposure to *A. fumigatus* conidia, contributing to COPD exacerbation during PA comorbidity (33). In addition, compromised immune surveillance increases susceptibility to fungal infections that trigger acute exacerbations (54). Macrophages derived from smokers and individuals with COPD exhibit impaired recognition and clearance of *A. fumigatus*, potentially due to altered receptor expression and blunted pathogen-responsive signaling, thereby increasing the risk of COPD–PA (55, 56). Collectively, macrophage dysfunction provides a mechanistic link between COPD pathobiology and susceptibility to PA.

AMs recognize fungal PAMPs primarily via CLRs and cooperate with humoral opsonins such as PTX3 and surfactant proteins (SP), thereby activating pro-inflammatory signaling, promoting phagocytosis, and favoring M1-skewed responses (51, 57). The diagnostic and prognostic value of PTX3 in patients with non-neutropenic IPA has garnered increasing attention (14). PTX3 is a soluble humoral pattern-recognition molecule that binds multiple ligands and thereby contributes to immune defense, inflammation, and diverse biological processes (58). Compared with CRP, PTX3 more faithfully reflects local inflammatory activity, and its covalently stabilized multimeric architecture is conducive to assay consistency and interpretability (59).Inflammatory dysregulation related to COPD promotes M2 anti-inflammatory polarization (60), reducing TNF-α, IL-1β, IL-6, and IL-8 secretion and impairing antifungal defense.

Neutrophils are key effector cells in COPD pathogenesis and anti-*Aspergillus* defense. Neutrophil accumulation directly depends on IL-6, IL-8, and CXC chemokine recruitment (61, 62). As noted, epithelial SYK/CARD9–NF-κB signaling drives neutrophil recruitment during *Aspergillus* pneumonia through inflammatory factor release, including IL-6 and IL-8 (38, 63). Furthermore, the neutrophil interleukin-1 receptor (IL-1R) regulates cell-autonomous survival during inflammation by modulating apoptosis and pro-survival pathways (64). Neutrophils execute intracellular and extracellular antifungal functions. Intracellular killing involves NADPH oxidase (NOX)-generated reactive oxygen species (ROS) (65). Extracellular killing is mediated by neutrophil extracellular traps (NETs) and antibody-dependent cellular cytotoxicity (ADCC) (65). ADCC requires antibody binding to Fc receptors (FcRs) on neutrophils, but the evidence regarding the mechanism of ADCC in the airways of COPD–PA patients remains limited, with research primarily focusing on NETs.

*Aspergillus* induces the formation of NETs, which entangle conidia and limit fungal dissemination (66). In COPD, neutrophil dysfunction can generate aberrant NETs that accumulate in the airway and form fibrin-rich networks, thereby amplifying chronic inflammation and tissue injury (67). Moreover, excessive release of neutrophil elastase in COPD degrades immunoglobulins and complement components, undermining opsonization and the clearance of *A. fumigatus*; this constitutes a mechanism of immune evasion by *A. fumigatus* (68).

**Figure 1:**
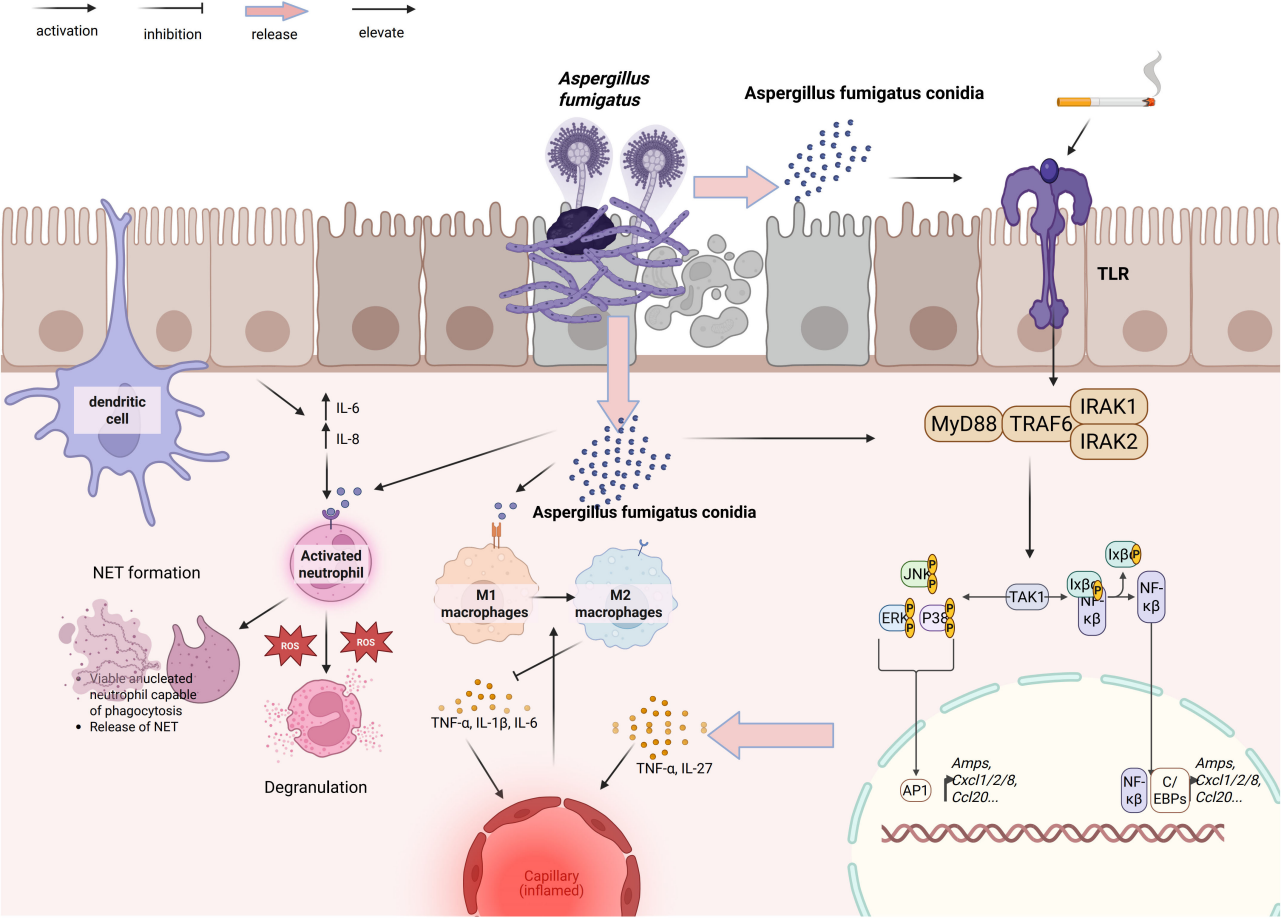
Epithelial–myeloid responses to *A. fumigatus* in COPD. Conidia activate epithelial TLR–MyD88–IRAK1/2–TRAF6–TAK1, triggering MAPK modules JNK/ERK/p38 and NF-κB/AP-1/C/EBPs to induce AMPs and chemokines CXCL1/2/8 and CCL20. DCs sample fungal material; recruited neutrophils degranulate, generate ROS, and form NETs. Macrophages polarize to M1 (TNF-α, IL-1β, IL-6) or M2 (TNF-α, IL-27). These circuits promote fungal control yet amplify airway and microvascular inflammation in COPD–PA. Arrowheads denote activation/flow; blunt bars denote inhibition; pale arrows indicate release/elevation.

**Figure 1** depicts the initial host–pathogen interface, illustrating how innate immune cells convert fungal recognition into inflammatory and antifungal responses and thereby couple early containment to downstream adaptive immunity.

### 2.3 Adaptive immunity: the final line of host defense

Dendritic cells (DCs) bridge innate and adaptive immunity and constitute a central arm of antifungal defense against *A. fumigatus (* 65). DCs translate innate pathogen recognition into programmed T-cell responses (69). Adaptive immunity is centered on T cells: T cells comprise CD8^+^ cytotoxic T lymphocytes (CTLs) that mediate targeted cytolysis, whereas CD4^+^ T helper cells differentiate into T helper 1 (Th1), T helper 2 (Th2), T helper 17 (Th17), and regulatory T (Treg) lineages. Through their cytokine networks, these subsets condition the airway microenvironment, calibrating the balance between pathogen clearance and immunopathology. This immunologic set-point, in turn, influences fungal burden, the risk of acute exacerbations, and the progression of structural lung injury.

CTLs, a CD8^+^ T-cell subset that recognizes and eliminates infected or transformed cells via apoptosis, are central to antiviral and antitumor immunity (70). Upon priming by dendritic cells via antigen presentation on MHC class I with costimulatory signals, and by Th1-derived cytokines such as interferon-γ (IFN-γ), naïve CD8^+^ T cells become activated and undergo clonal expansion (71). Activated CTLs induce target cell death predominantly through perforin–granzyme–dependent cytolysis (72).

DCs also orchestrate T-helper differentiation. Depending on the cytokine milieu, DCs activate naive CD4^+^ T cells and drive their differentiation into distinct effector lineages (e.g., Th1, Th2, Th17) or Treg (73). The cytokine milieu is partially determined by morphological forms of *A. fumigatus* and its cell-wall components recognized by DCs (74). CD4^+^ T lymphocytes amplify adaptive immunity by providing B-cell help and by licensing and enhancing CD8^+^ T-cell responses, whereas Treg restrain these processes, thereby modulating the magnitude and quality of inflammation (75).

Th1 and Th2 cells represent two principal subsets of CD4^+^ T cells: Th1 cells principally secrete IFN-γ and IL-2, coordinating cellular immunity, whereas Th2 cells produce IL-4, IL-5, and IL-13, thereby modulating humoral and type-2 inflammatory responses (76). Dysregulated CD4^+^ T-cell differentiation in COPD drives immune imbalance, with elevated Th1/Th2 ratios promoting persistent inflammation, airway remodeling, and emphysema (77). DCs stimulated by conidia of *Aspergillus* produce IL-12, which drives Th1 differentiation and induces IFN-γ production (78). Allergic manifestations induced by *Aspergillus*, such as ABPA, predominantly trigger Th2 responses, characterized by increased production of IL-4, IL-5, and IL-13 but reduced IFN-γ (79). IL-5 promotes eosinophil differentiation and chemotaxis, while IL-4 and IL-13 are involved in IgE production (79).

Accordingly, the Th1/Th2 axis serves as a central immunologic rheostat. Th1 responses driven by IFN-γ are associated with favorable anti-*Aspergillus* outcomes. However, when the Th1/Th2 balance shifts upward and IFN-γ signaling remains persistently elevated—particularly during acute exacerbations or in the presence of concurrent infection—COPD airway inflammation and tissue injury can worsen (80). Therefore, any use of exogenous or induced IFN-γ should be explored only as a context-specific adjunct to immunomodulatory therapy and evaluated cautiously in rigorously designed clinical trials (45). By contrast, Th2 polarization driven by IL-4, IL-5, and IL-13 is closely linked to ABPA, elevated IgE, eosinophilic airway inflammation, and mucus plugging, which exacerbate airflow obstruction and worsen prognosis. Across COPD cohorts, sensitization to *A. fumigatus* correlates with poorer lung function and more frequent exacerbations (prevalence≈13%; FEV_1_% predicted: 39% vs 51%, p=0.01), suggesting that heightened Th2 reactivity in COPD undermines effective antifungal immunity and amplifies allergic inflammation (81). The 2024 ERS/ISHAM guidelines identify COPD as a predisposing condition for ABPA (3).

Beyond the Th1/Th2 axis, the Th17/Treg axis constitutes a key arm of the immune processes underlying COPD–PA. Th17 cell differentiation depends critically on IL-6 and IL-23 (82). In fungal infection, CD4^+^ T cells binding DCs via Dectin-1 activate the Syk/CARD9 pathway, inducing IL-1β, IL-6, and IL-23 production that promotes Th17 differentiation and activation (83). Th17 cells secrete IL-17A and IL-17F, which enhance neutrophil recruitment and promote fungal clearance. While Th17 cells mediate crucial fungal clearance, dysregulated activation causes lung injury and chronic inflammation (84). COPD-derived pulmonary T cells demonstrate Th17 polarization (85); these cells exert pro-inflammatory effects via IL-17A, IL-17F, and IL-22 release (86).

In COPD–PA, whether Th17 cells exacerbate or mitigate disease progression remains debated. An analysis of a U.S. national inpatient database showed that, among admissions for acute exacerbations of COPD (AECOPD), in-hospital mortality was 14.5% in patients with IPA versus 3.6% in those without (5). Expert consensus recommends voriconazole as the preferred first-line agent for the initial treatment of COPD–IPA (87). Overall, the evidence indicates that invasive fungal infection is the principal driver in COPD–IPA, and timely antifungal therapy is critical (8). Accordingly, during the acute phase, the antifungal activity of the Th17 response appears to predominate, whereas COPD-related chronic inflammation is likely secondary. In a COPD–IPA mouse model, Geng et al. reported that Th17 cells failed to mount an effective response to pulmonary *Aspergillus* infection in the COPD setting, likely due to COPD-associated T-cell exhaustion (87). Lycan et al. reported that, despite the chronically inflamed milieu of COPD, T cells exhibit exhaustion-like dysfunction with upregulated immune checkpoints such as PD-1. This observation helps explain heightened susceptibility and mortality and argues against indiscriminate inhibition of the IL-17 pathway. Current work is concentrated on COPD–IPA; evidence regarding Th17 in CPA and ABPA is limited and requires more rigorous study.

Th17 cells and their signature cytokine IL-17 function as a double-edged sword. Their regulation is complex and is shaped by fungal immune evasion and activation strategies, as well as cigarette smoke exposure (74, 88, 89). Cell-wall components of *A. fumigatus* differentially regulate TLR2- and TLR4-dependent pathways; notably, α-glucan constrains IL-6 production via TLR2/TLR4 signaling, thereby dampening Th17 responses as an immune-evasion mechanism (74). *A. fumigatus* triggers Th1 and Th17 responses in mice via the TLR–MyD88 and Dectin-1 pathways, respectively; Dectin-1 recognition of BDG is a key driver of the Th17 response (88). Exposure to cigarette smoke extract increased IL-17 production by approximately 3–4-fold within hours (89).

Tregs exert anti-inflammatory effects through multiple pathways, notably the production of IL-10 and TGF-β, and CTLA-4–mediated, cell-contact–dependent inhibition of leukocyte function (90). Upon airway exposure to *A. fumigatus*, peripheral-blood *A. fumigatus*–specific Tregs undergo marked expansion, which is considered a mechanism that restrains Th2-dominant allergic responses (91). Tregs restrain excessive Th2 activity, thereby maintaining balance along the Th1/Th2 axis and sustaining Th1-mediated antifungal defense; they also curb dysregulated Th17 responses (92). This counter-regulatory architecture is advantageous in principle, preserving antifungal competence while limiting inflammation driven by Th2- or Th17-pathway hyperactivation. However, in COPD, Kalathil et al. reported that functionally suppressive Tregs and exhausted PD-1^+^ T cells contribute to effector T-cell dysfunction (93). Such T-cell dysfunction impairs fungal clearance, while inflammatory mediators such as IL-17 remain elevated (94), creating an immune milieu that favors the coexistence of persistent fungal burden and structural airway damage in COPD–PA.

B cell-mediated immune responses primarily generate antibodies crucial for pathogen neutralization and immunological memory (92). However, antigen-stimulated B lymphocytes promote pulmonary inflammation and damage through autoantibodies, cellular components, extracellular matrix proteins, and immune complex formation (92); they additionally enhance cellular immunity by functioning as antigen-presenting cells (APCs) (95). B lymphocytes producing IL-6 are classified as effector B cells (B-effs), while IL-10-producing cells are regulatory B cells (Bregs) that maintain immune homeostasis by preventing excessive inflammation and tissue damage (96). Peripheral blood lymphocytes in COPD show significantly reduced Breg counts and elevated B-eff/Breg ratios, causing adaptive immunity imbalance (97). DCs process *A. fumigatus* antigens and present them to naive T cells, activating Th1 cells and initiating targeted immune responses. Th1-derived cytokines—particularly IFN-γ—promote B-cell class switching to IgG production (98). The Th2-mediated IgE production mechanism was previously detailed. This provides the pathophysiological rationale for employing IgG and IgE as diagnostic markers.

Mucosal B cells produce secretory IgA (sIgA) that mediates immune exclusion at the airway surface. In COPD, epithelial barrier dysfunction together with focal sIgA deficiency facilitates adhesion and colonization of *Aspergillus* conidia. In parallel, reduced abundance or impaired function of regulatory Bregs diminishes regulatory control over *Aspergillus*-driven allergic inflammation. Asp IgG reflects sustained antigen exposure and is often markedly elevated in CPA (2, 99). Th2-driven class-switch recombination to IgE is the central mechanism of *Aspergillus*-related allergic disease, typified by ABPA. Allergen-specific IgE (and total IgE) is directly associated with eosinophilic inflammation, mucus impaction, and an increased risk of acute exacerbations (100). These pathophysiological links underpin the use of these antibodies as potential immunologic markers.

**Figure 2:**
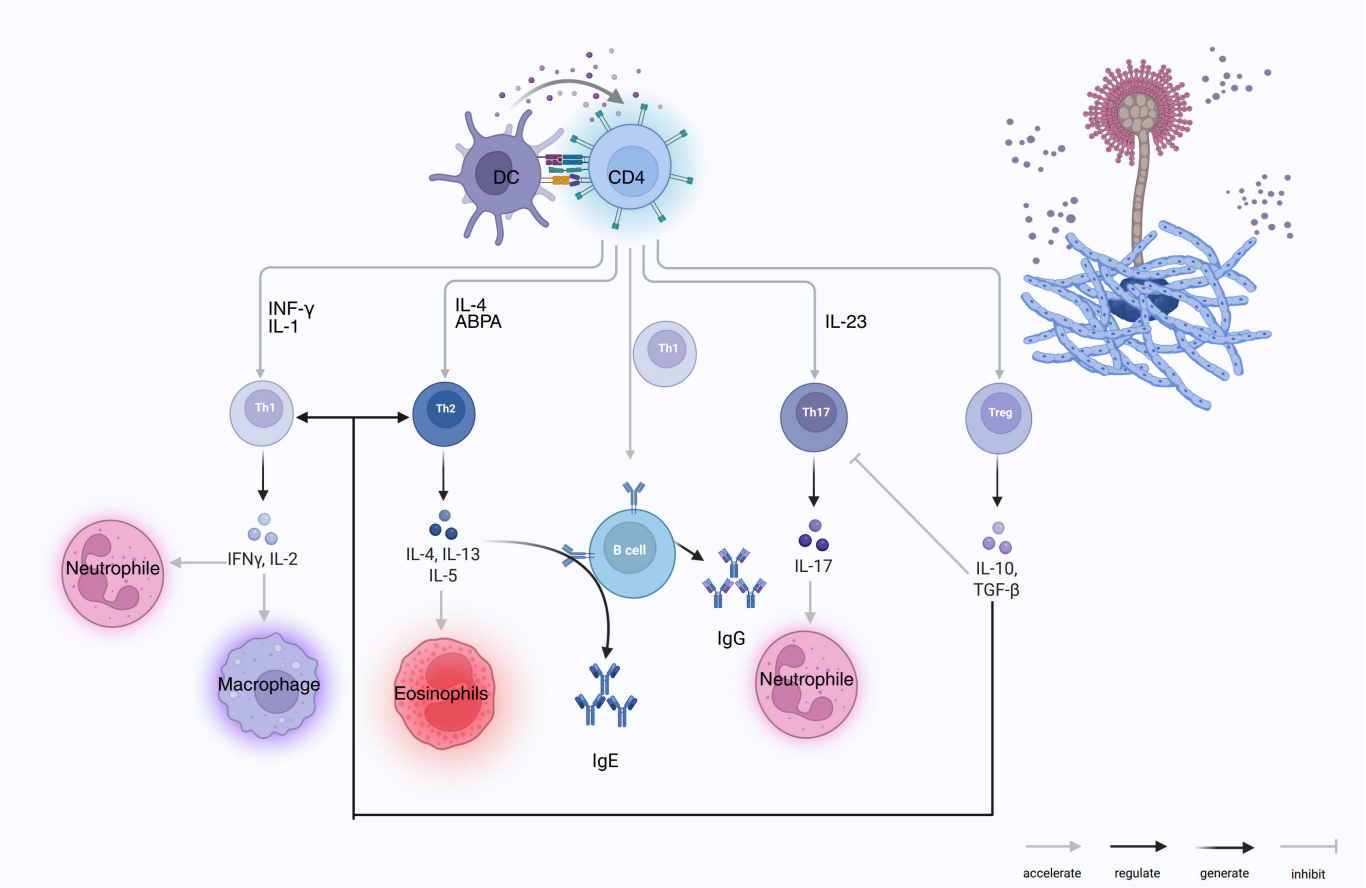
DC-CD4 polarization to Th1/Th2/Th17/Treg and effector outputs in COPD–PA. DCs present *A. fumigatus* antigens to CD4 T cells and drive lineage programs: Th1 (IFN-γ, IL-2) activates macrophages and supports neutrophils; Th2 (IL-4, IL-13, IL-5) induces eosinophilia and B-cell IgE class switching, the hallmark of ABPA; Th17 (IL-23→IL-17) recruits/activates neutrophils; Treg (IL-10, TGF-β) inhibits Th1/Th17 and restrains B-cell effector functions. With T-cell help, B cells generate IgG and IgE. Arrow keys: accelerate/regulate/generate/inhibit.

**Figure 2** summarizes how DCs sensing of *Aspergillus* instructs CD4^+^ T-cell lineage commitment (Th1, Th2, Th17, and Treg). These subsets, in turn, activate macrophage, neutrophil, and eosinophil effector programs and drive B-cell class-switch recombination to IgG and IgE, whereas Treg-derived IL-10 and TGF-β restrain excessive inflammation.

## 3 From disease trajectory and immune phenotypes to biomarker candidates

Building on the preceding overview of shared immune mechanisms in COPD–PA, it is clear that its clinical subtypes exhibit distinct immunologic profiles. In COPD–IPA, clinical presentations often mirror an acute exacerbation, with fever, cough, and dyspnea, thereby mimicking bacterial infection and complicating timely diagnosis (51). Among patients with AECOPD, concomitant IPA is associated with higher rates of cough, purulent sputum, hemoptysis, and fever compared with non-IPA cases (101). Early in the disease course, infection may remain confined to the tracheobronchial compartment, with vascular invasion emerging at later stages (102, 103). Accordingly, molecules engaged early in the airway immune response, such as GM, are best sampled from bronchoscopic specimens for early diagnosis, whereas serum measurements can assist in tracking disease progression. Consistent with this, bronchoalveolar lavage fluid (BALF) GM generally outperforms serum GM diagnostically (1, 104, 105), while serum GM may carry prognostic information (106). This perspective is necessarily incomplete: acute-phase reactants such as CRP may contribute to PA diagnosis, albeit with limited specificity (16). As the host response evolves, more specific antibodies and cytokines may accumulate and serve as adjunctive markers for diagnosis and longitudinal assessment.

COPD–CPA typically follows a chronic, progressive course, characterized by chronic cough, sputum production, fatigue, weight loss, and dyspnea; some patients remain asymptomatic for prolonged periods (51). According to ERS/ESCMID guidelines, CPA is categorized by chronicity, imaging, and mycologic evidence into simple aspergilloma, chronic cavitary CPA (CCPA), chronic fibrosing CPA (CFPA), *Aspergillus* nodule, and subacute invasive aspergillosis (SAIA) (2). Although CPA elicits immune responses, overall immunity, particularly cell-mediated immunity, is considered suboptimal (107), suggesting that cell-mediated effector products (e.g., IFN-γ) may be altered in CPA (2), with additional complexity introduced by underlying COPD. Sustained antigen exposure can drive persistent elevations of antibodies and cytokines beyond cell-mediated immunity, including IL-1, IL-6, and IL-8, which are amenable to serum-based investigation. ABPA features a type-2–skewed immune profile—elevated IL-4, IL-5, and IL-13, eosinophil activation, and high-titer allergen-specific IgE (79). These hallmarks naturally orient the search for COPD–ABPA immune markers.

Because COPD lacks pathogen-specific immune signatures, biomarker discovery from a COPD-only vantage is difficult (108, 109); however, mapping the distinct pathobiology and immune profiles of IPA, ABPA, and CPA reveals plausible biomarkers across these subtypes.

## 4 Immune markers

Based on extant evidence, we categorize immune biomarkers for COPD–PA into three tiers: (i) Experimentally Validated, (ii) Mechanistically Promising, and (iii) Requiring Further Investigation, reflecting current research maturity in this domain. Experimentally Validated biomarkers meet at least one of the following criteria: supported by international guidelines or expert consensus (such as ECCMID/ERS) and/or validated in COPD–PA–specific clinical cohorts. Mechanistically Promising biomarkers exhibit compelling evidence of involvement in COPD and aspergillosis pathways, demonstrate potential as standalone diagnostic indicators, but lack validation in dedicated COPD–PA cohorts. Requiring Further Investigation encompasses biomarkers with contradictory roles in COPD versus aspergillosis pathogenesis, necessitating mechanistic clarification prior to clinical application.

### 4.1 Experimentally validated

#### 4.1.1 GM and BDG

GM and BDG are key fungal cell wall components involved in multiple immune processes. Both are included as diagnostic markers in aspergillosis clinical practice guidelines (104, 110). Serum and BALF GM plus serum BDG serve as IPA diagnostic indicators, with BALF GM showing superior reliability (104). A four-year multicenter study demonstrated 88.9% sensitivity for BALF GM (ODI ≥0.5) in COPD-associated IPA diagnosis versus 11.1% for serum GM (ODI ≥0.5) (111). An earlier study evaluating GM in COPD-associated IPA identified an optimal BALF GM cutoff of approximately 0.8 (ODI), with 88.9% sensitivity and 100% specificity (105). However, frequent BALF sampling is contraindicated in comorbid or respiratory-impaired COPD patients (112), making serum testing a more feasible clinical alternative. Expert consensus endorses BALF GM positivity as microbiological evidence for CPA diagnosis in COPD (15). Salzer et al. demonstrated superior GM test diagnostic performance in COPD patients with CPA versus isolated CPA cases (113). Evidence indicates that GM has poor diagnostic performance for ABPA (114), and clinical practice guidelines explicitly discourage its use as a diagnostic tool (3); therefore, its utility in COPD–ABPA is unlikely to be favorable.

Taken together, GM shows favorable diagnostic performance in COPD–IPA and COPD–CPA. In practice, “serum GM” refers to the galactomannan index (GMI) measured in serum. In COPD–IPA, GMI also functions as a prognostic marker, particularly among AECOPD patients for whom invasive sampling is not feasible. In 153 acute AECOPD patients with IPA, positive GMI during initial ICU admission week correlated with poorer outcomes including reduced 28-day survival (106). These findings position serum GM as a prognostic biomarker for IPA in AECOPD. Multiple studies define serum GM positivity as GMI ≥ 0.5, whereas Yoshimura et al. regarded GMI ≥ 0.7 as a high level significantly associated with severe AECOPD and respiratory mortality (106, 115). Collectively, these observations support a screening threshold of GMI 0.5–0.7, to be interpreted alongside imaging and complementary biomarkers.

The diagnostic performance of BDG in IPA patients is suboptimal (116), so its use for detecting IPA in COPD patients is not regarded favorably. In a study involving 11 COPD patients with concomitant IPA, BDG testing was performed on 5 patients, with 3 testing positive (117). Potential explanations for BDG’s suboptimal performance include reduced specificity compared with GM because BDG is a ubiquitous fungal cell-wall component across many species, environmental glucan contamination, and interference from intravenous immunoglobulin (118). Clinical guidelines recommend combining BDG with GM to improve diagnostic performance (104). Reports on combined GM and BDG testing for diagnosing COPD–PA are limited. In a prospective single-center study (October 2006–November 2008) of 261 hospitalized AECOPD patients, dual-positive serum GM and BDG achieved 98.8% specificity—useful when BALF is not feasible—although the number of events was small (119). Hsu AJ et al. evaluated combined GM and BDG testing for invasive aspergillosis in immunocompromised hosts. Relative to GM alone, a both-positive rule markedly reduced sensitivity but yielded very high specificity, whereas an either-positive rule increased sensitivity with minimal loss of specificity (118). By extrapolation to COPD–PA, an either-positive GM/BDG approach may maximize sensitivity for early screening—particularly when BALF is not feasible—whereas a both-positive rule is preferable for confirmation to minimize false positives.

Although ibrexafungerp—a novel BDG-targeting antifungal—shows broad anti-*Aspergillus* activity and has shown efficacy in preclinical and clinical studies (120, 121), evidence in COPD patients with IPA remains limited. Multicenter randomized trials are needed to validate its utility in this population. Therefore, both BALF and serum GM represent valuable immune biomarkers for the IPA in COPD. BALF GM is primarily diagnostic, whereas serum GM provides greater prognostic value. In clinical practice, a combined GM–BDG testing strategy may be considered.

#### 4.1.2 IgG and IgM

Plasma Asp IgG is highly informative for diagnosing IPA in non-neutropenic patients (17, 122). Very high titers (>150 AU/mL) achieve ≥95% specificity. Using an optimal cutoff of 56.6 AU/mL, the sensitivity and specificity are 77.8% and 63.9%, respectively. In non-neutropenic IPA, its diagnostic performance exceeds that of serum GM but is slightly inferior to BALF GM (17). In the COPD subgroup, an Asp IgG threshold of approximately 72 AU/mL yields 73% sensitivity and 72% specificity, supporting Asp IgG as a blood-based alternative when bronchoscopy is impractical (17). Asp IgG can be measured by ELISA and, more recently, by a lateral flow assay (LFA). An “either positive” strategy that combines the IgG LFA with BALF GM or with sputum culture or serum GM markedly increases sensitivity (87.5 percent and 85.0 percent, respectively) while maintaining high specificity, indicating a useful noninvasive adjunct for patients who cannot undergo bronchoscopy (122). Comparative and combined use of GM, Asp IgG, and BDG may represent a promising direction for future research. Experts also recommend using Asp IgG as microbiological evidence for the diagnosis of CPA in COPD patients (15). Despite its well-established diagnostic performance, in a prospective multicenter cohort of non-neutropenic IPA (including a COPD subgroup), plasma Asp-IgG positivity was not associated with 90-day mortality; Asp-IgG levels did not differ between survivors and non-survivors, and Asp-IgG did not emerge as an independent prognostic factor in multivariable analysis (14). In a cohort of 59 patients with CPA (30 with COPD), regression analysis identified Asp IgG as a predictor of mortality (123).

Asp IgG and total serum IgE have been included in the diagnostic guidelines for ABPA (3). A retrospective study that analyzed clinical data from 251 bronchiectasis patients found significantly higher serum total IgE and Asp IgG levels in ABPA patients than in controls (124). Research has shown that IgE levels are elevated in AECOPD patients with *Aspergillus* colonization compared to those without (125). While the current utility of IgG and IgM is predominantly diagnostic, their prognostic value for disease progression in COPD-associated pulmonary aspergillosis warrants prospective validation.

#### 4.1.3 IL-1β

IL-1β, a key pro-inflammatory factor in COPD and PA. *Aspergillus* β-glucan and other PAMPs activate NF-κB via Dectin-1/CARD9 and TLR/MyD88 pathways, inducing IL1B transcription; subsequently, the NLRP3 inflammasome activates caspase-1 to process pro-IL-1β into its mature form (126). In airway models of aspergillosis, IL-1 receptor signaling sustains neutrophil survival and preserves antifungal activity (127). In COPD airways, cigarette smoke drives NLRP3–GSDMD-dependent IL-1β release, thereby amplifying airway inflammation (128). Analogous to IL-17, IL-1β contributes to antifungal defense yet exacerbates airway injury. Although macrophages in COPD may skew toward an M2 phenotype and dampen inflammation by reducing IL-1β (60), recent murine and cellular COPD models show that *A. fumigatus* exposure markedly elevates TNF-α, IL-1β, IL-6, and IL-33 (33).

Research on IL-1β has focused mainly on CPA, and its diagnostic performance for COPD–CPA appears suboptimal. One study suggested that the sensitivity of IL-1β, IL-6, and IL-8 as diagnostic biomarkers for CPA is higher in patients without COPD than in those with COPD (113). Nonetheless, IL-1β holds promise for monitoring and prognostic assessment in COPD–CPA. Serum IL-1β levels >2 pg/mL are associated with poorer outcomes in patients with CPA (129). In a cohort of 88 newly diagnosed CPA patients, AECOPD and active tuberculosis were associated with heightened pro-inflammatory responses and elevated IL-1β levels; an IL-1β concentration >20.3 pg/mL was defined as “high” (130). In the AECOPD–CPA subgroup (n=44), the high-IL-1β group exhibited greater disease activity, including more cavities (27/44 vs 13/44) and aspergillomas (25/44 vs 11/44); notably, IL-1β levels declined after surgical resection of lesions. Additionally, the administration of recombinant IL-1 receptor antagonist (IL-1Ra) reduces the pro-inflammatory Th17-mediated responses caused by *Aspergillus* infections, thereby improving prognosis (87). In corticosteroid-treated immunosuppressed mice with *Aspergillus* infection, IL-1Ra reduces lung tissue damage, thereby attenuating hypoxia (131). IL-1Ra thus represents a potential adjunct therapy for COPD–PA. Evidence for IL-1β in COPD–PA derives primarily from studies of CPA. Going forward, multicenter prospective cohorts enrolling COPD–CPA cases alongside COPD controls are needed, with methodological harmonization to define robust thresholds and enable external validation. In parallel, COPD–PA animal models should be developed to assess the safety and efficacy of IL-1Ra-based immunotherapy.

#### 4.1.4 IL-6 and IL-8

IL-6 and IL-8 are key cytokines in COPD and PA that mediate neutrophil recruitment. Their production and regulation in COPD–PA have been described earlier (33, 60, 63, 83, 113). In a prospective cohort of 106 patients with COPD, elevated IL-6 and IL-8 levels served as adjunctive diagnostic markers for IPA. For serum IL-6, the sensitivity and specificity were 74.32% and 81.25% at a cutoff of 92.82 pg/mL; for BALF, the corresponding values were 68.92% and 71.88% at 229.4 pg/mL. For serum IL-8, the sensitivity and specificity were 83.78% and 81.25% at 93.46 pg/mL; in BALF, they were 85.14% and 75.00% at 325.4 pg/mL, indicating marginally better diagnostic performance in serum than in BALF (132). Additionally, IL-6, IL-8, and TNF-α levels help differentiate patients with CPA-TB from those with TB alone (133). Huang et al. further showed that pro-inflammatory mediators—including TNF-α, IL-6, and IL-8—increase with worsening CPA in both univariate and multivariate analyses (129). Whether IL-6 and IL-8, akin to IL-1β, can serve as prognostic markers for disease progression in COPD–CPA remains to be determined. A case report of invasive aspergillosis occurring after tocilizumab (anti–IL-6R) therapy in a patient with COVID-19 cautions against indiscriminate extrapolation of anti-IL-6/IL-8 strategies to COPD–PA (134), and many questions remain unresolved.

#### 4.1.5 IL-17

He et al. reported that in non-neutropenic IPA, including a subgroup with COPD, plasma and BALF IL-17 demonstrated useful diagnostic performance, with sensitivity exceeding that of GM but lower specificity (94). Optimal cutoffs were identified as 12.02 pg/mL for plasma IL-17 (sensitivity 72.6%, specificity 69.4%) and 21.32 pg/mL for BALF IL-17 (sensitivity 81.2%, specificity 72.6%) (94). Geng et al. established an IL-17A gene knockout COPD–IPA mouse model and found that its *Aspergillus* fungal load was nearly twice that of the regular COPD–IPA mouse model, suggesting that IL-17 contributes to antifungal defense (87). Given the predominantly antifungal activity of IL-17 observed in experimental models, IL-17–based adjunctive strategies for COPD–IPA merit cautious evaluation rather than direct supplementation.

#### 4.1.6 Pentraxins

Pentraxins, defined by a conserved pentraxin domain, comprise short pentraxins (such as CRP) and long pentraxins (such as PTX3). CRP serves as a biomarker for disease severity and prognosis in COPD and PA (123, 135). In a cohort of hospitalized patients with COPD (GOLD III–IV), a day-1 CRP level >1.29 mg/L was identified as the optimal diagnostic cutoff for COPD-associated IPA, with sensitivity 91.2% and specificity 57.7%. Pairwise combinations further improved discrimination, such as CRP plus ESR and CRP plus LDH (16). CRP may have dual utility for monitoring and diagnosing IPA in patients with COPD. However, coexisting bacterial infections—common in COPD-associated IPA—can confound the interpretation of CRP (136, 137), necessitating further validation of its standalone or combined diagnostic utility.

PTX3, an acute-phase pattern recognition receptor (138), demonstrates diagnostic value in non-neutropenic IPA (139). In a multicenter prospective cohort of non-neutropenic IPA that included a COPD subgroup, Sun et al. identified 90-day mortality stratification thresholds of plasma PTX3 ≥7.11 ng/mL (AUC 0.82; sensitivity 82.8%; specificity 73.4%) and BALF PTX3 ≥4.29 ng/mL (AUC 0.76; sensitivity 81.4%; specificity 67.1%) (14). Recombinant PTX3 has shown therapeutic efficacy against aspergillosis in murine and rat IPA models; however, human studies are still lacking (140). Novel anti-*Aspergillus* agents continue to emerge in the pharmaceutical pipeline.

### 4.2 Mechanistically promising

#### 4.2.1 IL-5

ABPA, a type 2 inflammatory disease, is characterized by elevated IL-5 (141). Biologics targeting IL-5 and its receptor α (IL-5Rα) may benefit patients with COPD; in selected eosinophilic COPD populations, these agents significantly reduce annual exacerbation rates and serious adverse events (142, 143). Based on this, attempts have been made to treat ABPA with biologics, including omalizumab (anti-IgE), mepolizumab (anti-IL-5), benralizumab (anti-IL-5R), dupilumab (anti-IL-4Rα), and tezepelumab (anti-TSLP) (144). According to the ISHAM-ABPA guidelines, biologics are not recommended as first-line therapy for acute ABPA. Initial treatment is oral prednisolone or itraconazole, and combination therapy is considered only for relapses (3). Direct evidence of efficacy of anti-IL-5/IL-5Rα in COPD–ABPA is lacking. In ABPA with comorbid asthma, real-world and retrospective studies report that anti-IL-5/anti-IL-5Rα monoclonal antibodies reduce exacerbation frequency, decrease oral corticosteroid requirements, and improve lung function (145). Therefore, large-scale randomized trials with extended follow-up are required to evaluate the efficacy of biologics in COPD–ABPA. Key endpoints should include annualized exacerbation rate, cumulative oral corticosteroid exposure, relief of mucus plugging, and radiologic improvement. Randomization should be stratified by blood eosinophil count and *Aspergillus* sensitization, with comparator arms including anti-IgE therapy and azole monotherapy.

#### 4.2.2 TARC

Thymus and activation-regulated chemokine (TARC) is associated with eosinophilic inflammation and IgE activation, and mechanistically aligns with the type-2 inflammatory axis of ABPA (146). In ABPA, Kozlova et al. reported markedly elevated serum TARC (median, 734.0 pg/mL) that decreased significantly after 12 weeks of itraconazole, paralleling improvements in FEV_1_ and reductions in total IgE, thereby supporting its use for monitoring antifungal treatment response (147). In COPD higher TARC concentrations are associated with rapid decline in lung function: a serum TARC ≥211 pg/mL identified rapid FEV_1_ decline (sensitivity 100%, specificity 26.8%), and TARC ≥335 pg/mL identified rapid %FEV_1_ decline (sensitivity 67.5%, specificity 55.4%), suggesting that TARC may serve as an independent predictor of accelerated deterioration (148). However, sensitivity/specificity thresholds in COPD–PA/ABPA require prospective validation. At present, TARC is best considered an exploratory companion marker.

#### 4.2.3 Th17/Treg

The potential immunologic roles of Th17 and Treg in COPD–PA have been outlined above. In a prospective cohort of acute respiratory distress syndrome (ARDS), a Th17/Treg ratio >0.79 predicted 28-day mortality (sensitivity 87.5%, specificity 68.1%) (149), which initially suggested potential prognostic utility for COPD–PA; however, closer appraisal tempered this expectation. In COPD, the Th17/Treg ratio is not uniformly elevated and varies by disease stage and sampling compartment (86). Even within the same PA subtype (ABPA), studies report opposing directions (79, 150), and differences across subtypes are likely—though direct evidence is limited. Given the added immunologic complexity of COPD with PA, other markers, such as IL-1β, may offer greater predictive value.

#### 4.2.4 SP-D

Surfactant protein D (SP-D) is a critical component of the pulmonary innate immune system. It mediates functions of innate and adaptive immune cells and participates in clearing apoptotic cells, allergens, and other harmful particles. Cigarette smoking promotes SP-D translocation from alveolar spaces into the bloodstream, establishing serum SP-D as a validated biomarker of smoke-induced pulmonary damage (151). One study indicated that serum SP-D levels are not associated with the development of COPD (152). Notably, serum SP-D demonstrates limited diagnostic or monitoring utility for ABPA exacerbations (153). Although SP-D demonstrated protective efficacy in immunosuppressed murine models challenged with *A. fumigatus* conidia, enhancing fungal clearance, the available evidence does not support SP-D as a diagnostic or prognostic biomarker in COPD–PA (154).

### 4.3 Requiring further investigation

#### 4.3.1 Immune receptors and signaling molecules

NF-κB, TLR2, TLR4, JNK, and Dectin-1 have been discussed in earlier sections as potential immunological biomarkers. Due to their established cross-reactivity within immune pathways, these molecules are frequently explored as therapeutic targets. However, the activation of these signaling molecules and immune receptors can serve a dual function: while they exert antifungal effects, they may also exacerbate airway inflammation. Taking the JNK pathway as an example, epithelial cells are stimulated by A. fumigatus conidia to activate the JNK signaling pathway, which facilitates fungal clearance (48) However, activation of the JNK pathway is also associated with epithelial damage and airway inflammation in COPD (46, 47). Moreover, evidence supporting their use as immunological biomarkers for COPD–PA remains limited, particularly in clinical cohorts. Most studies have primarily focused on their roles in immunomodulation and inflammatory cascades, with systematic evaluations of their biomarker potential still notably absent.

#### 4.3.2 IFN-γ

In COPD, heightened Th1 and Th17 activity drives IFN-γ production, mediating pulmonary inflammation and tissue damage (21). IFN-γ is a central antifungal cytokine that activates macrophages, augments antimicrobial-peptide expression, and promotes effector T-cell recruitment to coordinate neutrophil-mediated fungal clearance (63). In parallel, IL-12 signaling downstream of the TLR–MyD88 and Dectin-1/CARD9 axes drives Th1 differentiation, thereby upregulating IFN-γ production (40, 78). Notably, many cytokines—including IL-1β, IL-6, IL-8, IL-17, and IFN-γ—support antifungal defense yet concurrently exacerbate airway inflammation and tissue injury in COPD. However, a study observed that CPA patients exhibit defective production of IFN-γ and IL-17A (155). IFN-γ replacement therapy has also been included in CPA clinical treatment guidelines (50–60 μg subcutaneously, three times weekly) (2). ABPA involves predominant Th2 responses with concomitant Th1 suppression, inducing IFN-γ deficiency. Additionally, IPA is commonly observed in immunocompromised patients (156). Russo et al. observed that IFN-γ knockout mice exhibited increased susceptibility to IPA, and also noted a marked decrease in IFN-γ expression in severe IPA patients (157). IFN-γ replacement therapy may hold substantial promise for the treatment of PA, yet excessive IFN-γ can exacerbate COPD. This paradox complicates IFN-γ replacement therapy in COPD–PA: in the COPD-dominant phenotype, elevated IFN-γ contraindicates supplementation, whereas in the PA-dominant phenotype, IFN-γ therapy may be feasible but likely within a narrow therapeutic window. Accordingly, IFN-γ replacement should first be evaluated in COPD–PA animal models using dose-escalation designs to define an exposure–response threshold, followed by rigorously controlled studies in COPD–PA patients. **Table 1** summarizes the immunological biomarkers discussed in the preceding sections.

**Table 1:** Immune markers in COPD–IPA/CPA/ABPA: classification, key characteristics, and evidence.

Subtype	Classification level	Immune markers	Possible function/characteristics	Classification reason
COPD–IPA	**Tier1**	GM	• Early Diagnosis (105, 111) • Monitoring and Prognostic Assessment (106, 115)	• Supported by guidelines/consensus (1, 15, 110) • validation in COPD–PA-specific clinical cohorts (105, 106, 111, 115)
		BDG	• Combination with GM improves diagnostic performance (104, 119) •Target of novel antifungals (e.g., ibrexafungerp) (120, 121)	• Supported by guidelines/consensus (104, 110) • validation in COPD–PA-specific clinical cohorts (117, 119)
		ASP IgG	• Early Diagnosis (17) •Combination with GM improves diagnostic performance (17)	• Supported by guidelines/consensus (15) • validation in COPD–PA-specific clinical cohorts (17)
		IL-6 and IL-8	• Adjunctive tools for IPA diagnosis in COPD (prospective validation) (132)	• validation in COPD–PA-specific clinical cohorts (132)
		IL-17	• Early Diagnosis (94) •Critical for antifungal defense (nearly 2× fungal load in IL-17A-KO mice) (87)	• validation in COPD–PA-specific clinical cohorts/animal model (87, 94)
		CRP	• Monitoring and Prognostic Assessment (16) •Enhanced diagnostic accuracy when combined with ESR/LDH (16)	• validation in COPD–PA-specific clinical cohorts (16)
		PTX3	•Independent prognostic factor for non-neutropenic IPA mortality (14) •Therapeutic potential in preclinical models (140)	• validation in COPD–PA-specific clinical cohorts (14)
	**Tier2**	NA	NA	NA
	**Tier3**	IFN-γ	• Pro-inflammatory tissue damage in COPD (21) • Defective production in IPA (157)	• Contradictory evidence, requires clarification (21, 79, 155)
		NF-κB, TLR2, TLR4, JNK, Dectin-1	• Key signaling nodes in immune dysregulation (36–40, 42–47) • Antifungal effector function following activation (48) • Activation-dependent exacerbation of airway inflammation in COPD (46, 47)	• Contradictory evidence, requires clarification (46–48)
COPD–CPA	**Tier1**	IL-1β	•Monitoring and Prognostic Assessment (130)	•validation in COPD–PA-specific clinical cohorts (130)
		Asp IgG	• Early Diagnosis (15) •Monitoring and Prognostic Assessment (123)	•Supported by consensus (15) •validation in COPD–PA-specific clinical cohorts (123)
	**Tier2**	NA	NA	NA
	**Tier3**	IFN-γ	• Pro-inflammatory tissue damage in COPD (21) • Defective production in CPA (2, 155)	• Contradictory evidence, requires clarification (21, 79, 155)
		NF-κB, TLR2, TLR4, JNK, Dectin-1	• Key signaling nodes in immune dysregulation (36–40, 42–47) • antifungal effector function following activation (48) • activation-dependent exacerbation of airway inflammation in COPD (46, 47)	• Contradictory evidence, requires clarification (46–48)
COPD–ABPA	**Tier1**	ASP IgG and serum IgM	• Early Diagnosis (125)	• Supported by guideline (3) • validation in COPD–PA-specific clinical cohorts (125)
	**Tier2**	TARC	•Biomarker for ABPA diagnosis/monitoring (147) •Independent predictor of rapid lung function decline (FEV_1_) in COPD (148)	• Independent COPD and ABPA evidence (147, 148)
		IL-5	•Central to ABPA pathogenesis (141) •Anti-IL-5 biologics reduce exacerbations in eosinophilic COPD (142, 143)	• Independent COPD and ABPA evidence (142–144)
	**Tier3**	NF-κB, TLR2, TLR4, JNK, Dectin-1	• Key signaling nodes in immune dysregulation (36–40, 42–47) • antifungal effector function following activation (48) • activation-dependent exacerbation of airway inflammation in COPD (46, 47)	• Contradictory evidence, requires clarification (46–48)

## 5 Translational clinical applications of immune biomarkers

### 5.1 Early diagnosis

In COPD–PA, the usefulness of conventional microbiology and imaging for early recognition is limited by several factors, particularly the relative rarity in COPD patients of classical IPA radiologic signs such as the air-crescent sign and the halo sign (12, 13, 158, 159). Immune markers: (i) may serve as complementary evidence alongside standard microbiologic and radiologic assessments; (ii) may improve diagnostic accessibility when bronchoscopy or tissue biopsy is not feasible or is contraindicated; and (iii) may be applied in combination, whereby different panels and decision rules, including the “either-positive” and “both-positive” rules, yield distinct sensitivities and specificities, thereby informing clinical decision-making.

### 5.2 Monitoring and prognostic assessment

In COPD–PA, disease activity fluctuates in response to airway inflammation, acute exacerbations, bacterial co-infection, and the intensity of antifungal or adjunctive therapies, which may blunt or amplify conventional clinical signals. IPA and ABPA typically present acutely and are associated with poorer prognoses, whereas CPA generally follows a more indolent course (3, 158, 160). Immune markers: (i) may aid early risk stratification during acute episodes and thereby inform interventions that improve outcomes and survival; (ii) in chronic disease, may help delineate disease stage and support a biomarker-based staging framework for COPD–CPA; and (iii) if staging can be anchored to immune-biomarker profiles, it may enable more systematic, stage-specific therapeutic strategies while acknowledging interindividual variability. Taken together, integrating immune-biomarker readouts with clinical, radiologic, and microbiologic assessments may strengthen longitudinal monitoring and prognostic assessment across the COPD–PA spectrum.

Based on current studies evaluating the diagnostic and prognostic utility of immune markers in COPD- and non-neutropenic patient–associated IPA, we synthesized and summarized the relevant data (**Table 2**). Given the paucity of studies on COPD-associated CPA and ABPA—we did not present corresponding tables.

**Table 2:** Immune markers for early diagnosis and prognostic assessment of IPA in COPD/non-neutropenic patients: validated cutoffs, sensitivities and specificities.

Clinical application	Subtype	Immune markers	Cutoff	Sensitivity	Specificity	Reference
Early Diagnosis	(AE)COPD–IPA	GM (BALF)	ODI ≥0.5	88.9%	88.4%	(111)
		GM (BALF)	ODI ≥1.0	66.7%	96.2%	(111)
		GM (BALF)	ODI ≈0.8 (optimal)	88.9%	100%	(105)
		GM (Serum)	ODI ≥0.5	11.1%	96.2%	(111)
		Asp IgG (Serum)	≥72.0 AU/mL (COPD- subtype optimal)	73.1%	72.0%	(17)
		IL-6 (Serum)	92.82 pg/mL	74.32%	81.25%	(132)
		IL-6 (BALF)	229.4 pg/mL	68.92%	71.88%	(132)
		IL-8 (Serum)	93.46 pg/mL	83.78%	81.25%	(132)
		IL-8 (BALF)	325.4 pg/mL	85.14%	75.00%	(132)
		CRP (Serum)	1.29 mg/L	91.2%	57.7%	(16)
		GM+BDG (Serum and both positive rules)	ODI≥1.5 and BDG ≥80pg/mL	80%	98.8%	(119)
	non-neutropenic patients (including COPD)—IPA	IL-17A (Serum)	12.02 pg/mL (optimal)	72.6%	69.4%	(94)
		IL-17 (BALF)	21.32 pg/mL (optimal)	81.2%	72.6%	(94)
		GM (Serum)	ODI≥0.6 (optimal)	56.5%	87.7%	(94)
		GM (BALF)	ODI≥1.01 (optimal)	68.7%	91.9%	(94)
		Asp IgG (Serum)	≥56.6 AU/mL (optimal)	77.8%	63.9%	(17)
		GM+IL-17 (Serum and both positive rules)	ODI≥0.6 and 12.02 pg/mL	67.7%	83.1%	(94)
		GM+IL-17 (BALF and both positive rules)	ODI≥1.01 and 21.32 pg/mL	81.2%	83.7%	(94)
		IgG LFA+ BALF GM (either positive rules)	≥ 135 AU/mL	87.5%	85.0%	(122)
Monitoring and Prognostic Assessment	(AE)COPD–IPA	GMI (peak serum value within the first ICU week and prediction of 28-day outcomes)	GMI>0.483	51.5%	81.9%	(106)
	non-neutropenic patients (including COPD)—IPA	PTX3 (Plasma of 90-day non-survivors versus survivors)	PTX3 > 7.11 ng/mL	82.8%	73.4%	(14)
		PTX3 (BALF of 90-day non-survivors versus survivors)	PTX3 > 4.29 ng/mL	81.4%	67.1%	(14)

### 5.3 Immunotherapy

Traditional antifungal therapy is limited by toxicity and pharmacologic constraints: for example, amphotericin B is nephrotoxic (161); triazoles have extensive drug–drug interactions that necessitate therapeutic drug monitoring (TDM) (1–3, 110); and azole resistance in *Aspergillus* is increasingly reported across multiple regions, with resistance in invasive aspergillosis associated with higher mortality and diminishing the effectiveness of first-line azoles (162). Immune markers: (i) may be combined with antifungal agents to enhance therapeutic efficacy; (ii) may aid the management of refractory disease; and (iii) may help mitigate antifungal-related adverse effects.

### 5.4 Biomarker-guided algorithms and cutoffs for COPD–PA subtypes

Anchored in the tiered framework (**Table 1**) and the standardized cutoffs and reporting conventions (**Table 2**), the following subtype-specific recommendations translate biomarker evidence into bedside algorithms. For suspected COPD–IPA, prioritize testing BALF GM (optimal ODI ≥ 1.01; sensitivity 68.7%, specificity 91.9%) when bronchoscopic sampling is feasible and safe; otherwise, employ a serum-centered panel comprising GM (optimal ODI ≥ 0.6; 56.5%, 87.7%) with or without PTX3, IL-6, and IL-8. An any-positive rule supports early screening, whereas a dual-positive rule can serve for confirmation—for example, serum GM + BDG (ODI ≥ 1.5 and BDG ≥ 80 pg/mL; 80.0%, 98.8%) or GM + IL-17 (serum: 0.6 and 12.02 pg/mL; 67.7%, 83.1%; BALF: 1.01 and 21.32 pg/mL; 81.2%, 83.7%)—before initiating antifungal therapy that entails toxicity and resistance. For suspected COPD–CPA, base the diagnosis on Asp IgG (optimal ≥ 56.6 AU/mL; 77.8%, 63.9%; very high titers > 150 AU/mL achieve ≥ 95% specificity; COPD subgroup approximately 72 AU/mL yields 73%/72%) and incorporate IL-1β for activity and prognostic assessment; employ sequential retesting when clinically indicated. For COPD–ABPA, follow the guideline pathway centered on total IgE and Asp IgG; when feasible, consider TARC and IL-5 as activity markers or companion readouts to therapy. For longitudinal monitoring and prognostication in non-neutropenic patients (including COPD) with IPA, consider GMI (peak value in the first ICU week > 0.483; 51.5%, 81.9%) and PTX3 (plasma > 7.11 ng/mL; 82.8%, 73.4%; BALF > 4.29 ng/mL; 81.4%, 67.1%), alongside CRP and selected airway cytokines (such as IL-8: serum 93.46 pg/mL; 83.78%, 81.25%; BALF 325.4 pg/mL; 85.14%, 75.00%).

## 6 Discussion

This review synthesizes evidence on immune biomarkers in COPD complicated by IPA, CPA, and ABPA and advances a tiered, clinically oriented framework (**Table 1**). Tier 1 comprises GM, BDG, Asp IgG, IgE, CRP, PTX3, IL-1β, IL-17, IL-6, and IL-8; taken together, these biomarkers support diagnosis, with BALF GM as the anchor, and inform risk stratification and prognostication, with particular relevance of PTX3, the serum GMI, and IL-1β. Tier 2/3 encompasses candidates that are mechanistically grounded or promising yet require further validation, including IL-5 and TARC, as well as pathway nodes such as NF-κB, TLR2/4, JNK, and Dectin-1. The role of IFN-γ appears context-dependent and bidirectional, underscoring the need for clarification specifically in COPD–PA.

Pathophysiologically, the evidence is consistent with a proximal-airway to systemic spillover trajectory: in early disease, airway fungal burden and mucosal inflammation predominate, which explains the higher sensitivity of BALF GM and selected airway cytokines; as the disease progresses, serum readouts become more informative, such as the GMI, PTX3, and systemic inflammatory mediators. To enhance comparability and clinical translation, **Table 2** collates commonly used cutoffs and reporting conventions as practical reference points for future studies and pilot implementation.

A cross-study synthesis revealed recurring themes. Study-population heterogeneity was pervasive: many cohorts were not COPD–PA exclusive and enrolled non-neutropenic or non-immunosuppressed patients in whom COPD formed only a subgroup; small sample sizes, stringent exclusion criteria, and categorization bias (such as liberal assignment to “possible IPA”) hampered effect-size estimation and constrained the generalizability of thresholds (14, 17, 94). Assay platforms and workflows also differed, yielding non-uniform cutoffs; for example, serum GMI is commonly interpreted around 0.5–0.7 (106, 115). In the context of COPD–CPA, IL-1β thresholds of 2 pg/mL and 20.3 pg/mL have been reported for monitoring and prognostication; the former signals poorer outcomes and the latter defines a higher activity tier, underscoring that cutoff selection depends on study objectives (129, 130). Combination rules behaved predictably: dual-positive panels, such as GM plus BDG or GM plus IL-17, increased specificity at the cost of sensitivity and thus favored confirmation, whereas any-positive panels maximized sensitivity with only modest specificity loss, supporting early screening when BALF cannot be obtained promptly (94, 119). Evidence is skewed toward COPD–IPA, followed by COPD–CPA, with COPD–ABPA markedly underrepresented. Across the research pipeline, the field remains diagnosis-centric, then monitoring and prognostication, and only lastly treatment, reflecting an early emphasis on identifying high-sensitivity and high-specificity markers to enable timely antifungal therapy. Finally, cytokines act as a double-edged sword: they can aid fungal clearance yet aggravate COPD airway inflammation. We hypothesize that, during acute IPA, fungal infection is the primary driver whereas COPD airway inflammation is secondary—a provisional inference that aligns with portions of the literature. IFN-γ, immune receptors, and signaling molecules were highlighted in Tier 3 because IL-6, IL-8, IL-1β, and IL-17 already have clinical or preclinical support in COPD–PA, whereas these Tier-3 targets currently lack comparably robust evidence and remain exploratory.

Future research on COPD–PA should be organized around several themes. First, increase attention to COPD–CPA and COPD–ABPA, with parallel emphasis on immune biomarkers for risk stratification, prediction, and immunotherapeutic decision-making. Second, multicenter studies must be expanded to increase statistical power and external validity. Current COPD–PA studies typically enroll several dozen to two or three hundred participants; sample sizes tend to be larger in IPA—such as 153 and 261 cases (106, 119)—are commonly in the tens for CPA (123, 130), and dedicated reports on COPD–ABPA remain scarce. Future work should prioritize multicenter designs; Third, combinatorial application should become standard practice: single immune biomarkers have inherent limitations; antibiotic exposure can produce false-positive GM results (163), and corticosteroid use together with COPD-related immune dysregulation diminishes the diagnostic utility of antibody testing for IPA in this population (164). Immune markers panels should be interpreted together with clinical findings, mycological results, and imaging. When appropriate, they can also be incorporated into machine-learning diagnostic models that use multiple predictors (165).
